# Continuous evolution of magnetic resonance imaging in multiple sclerosis

**DOI:** 10.1007/s00415-016-8099-y

**Published:** 2016-04-01

**Authors:** E. C. Tallantyre, N. P. Robertson

**Affiliations:** Department of Neurology, Institute of Psychological Medicine and Clinical Neuroscience, Cardiff University, Cardiff, UK

Since the introduction of MR imaging into routine clinical practice in the 1990s, it has become firmly embedded into the diagnostic criteria of multiple sclerosis (MS). Furthermore, serial MR brain imaging is increasingly being used to complement contemporary clinical assessment of MS disease activity. Clinical imaging in MS largely relies upon the detection of T2-hyperintense and T1-gadolinium enhancing lesions, as surrogate markers of foci of inflammatory demyelination in the white matter. Beyond the scope of conventional clinical imaging, advanced MR technology is now capable of detecting and quantifying pathology in tissue compartments previously thought to be unaffected in MS. The appreciation of microscopic damage within the “normal-appearing white matter” and grey matter of the brain and spinal cord has considerably advanced our knowledge of the pathophysiology of MS.

In this month’s journal club we describe three papers that relate to MR imaging in MS. This first paper describes the latest MR criteria for MS diagnosis. The second paper describes the imaging characteristics of the radiologically isolated syndrome and how they, along with clinical factors, relate to the subsequent onset of symptomatic demyelinating disease. The third paper uses advanced MR technology to uncover the regional pattern of cortical atrophy in MS and its relationship to white matter pathology and clinical disability.

## MRI criteria for the diagnosis of multiple sclerosis: MAGNIMS consensus guidelines

Many patients who present with clinical syndromes typical of inflammatory-demyelination in the CNS are found to have subclinical lesions on MRI scans. The detection of subclinical brain or cord lesions with typical morphology predicts a higher risk of subsequent conversion to MS. Indeed, since 2001 the diagnostic criteria of MS have permitted MR imaging to confirm the diagnosis of MS, either at baseline or after an interval in an individual with a typical clinical syndrome. The MR imaging criteria stipulate the conditions in which the requirements for dissemination of demyelination in space and time can be made in those who have experienced a single clinical attack or a progressive course from onset.

This paper describes the most recent refinement of the MR criteria used to define MS, based on the most contemporary imaging data and techniques. The guidelines are based on the findings of an international workshop, held in March 2015 under the auspices of MAGNIMS (a European collaborative research network that studies the use of MRI in MS). Recommendations are based on both expert opinion and literature review.

The main changes to existing MRI criteria relate to the definition of dissemination in space. The new criteria propose that the optic nerve is incorporated as one of five lesion locations (along with periventricular, infratentorial, cortical/juxtacortical and spinal cord) that can be used to define dissemination in space. The presence of lesions in two out of these five areas is deemed sufficient evidence to define dissemination in space. In all except the periventricular region, only one lesion is required to be present. These most recent guidelines suggest that at least three periventricular lesions should be required to establish dissemination in space at this location, in view of the low specificity of a solitary periventricular lesion. No distinction is required between lesions considered to be symptomatic or asymptomatic.

The criteria for dissemination in time remain unchanged: the detection of either one new T2 lesion or one new gadolinium-enhancing lesion after any interval, or the coexistence of enhancing and non-enhancing lesions at any time. In these new recommendations, interval spinal cord imaging is not recommended in asymptomatic individuals for clarification about dissemination in time as clinically silent cord lesions are recognised to be rare.

### Comment

These new criteria incorporate the most recent evidence and opinion in the field and in some ways they reflect a simplification of the older criteria (for example avoiding the clinical dilemma of whether a lesion is symptomatic, which can be challenging outside of the optic nerve and spinal cord). However, these new criteria may also be an unwelcome change for our reporting radiologists and for the minority of individuals whose diagnosis becomes altered, given that prospective validation of sensitivity and specificity in a longitudinal clinical cohort has not yet been demonstrated.

It is important to stress that these criteria only apply to those individuals who have a clinical presentation that is typical of MS. The MR criteria for dissemination in space and time could readily be met in individuals with certain other neurological conditions. So it is crucial to remember that the diagnosis of MS lies additionally in the exclusion of any better explanation for the symptoms. The new proposal to apply these MR criteria to individuals with a radiologically isolated syndrome (uncovered inadvertently when imaging for another reason) is aimed at facilitating a prompt diagnosis whenever symptoms typical of CNS demyelination occur, but it may also enhance our understanding of the pre-clinical phase of MS (see Kantarci et al. below).

Filippi et al. (2016) Lancet Neurol 15:292–303.

## Primary progressive multiple sclerosis evolving from radiologically isolated syndrome

Increasing use of MR scanning and improvements in MR technology have led to the relatively frequent identification of individuals who have incidental lesions in the brain or spinal cord. However, the concept of a “radiologically isolated syndrome” (RIS) of MS is a relatively recent phenomenon. First described in the literature in 2009, RIS describes the incidental finding of CNS lesions that have a morphology and location typical for inflammatory-demyelination (the latter is established using the Barkhof criteria), and which cannot be explained by another process, including cerebral small vessel disease. RIS has since been shown to convert to symptomatic MS in around a third of individuals over a 5-year period, with higher risk conferred by young age, male sex and spinal cord lesions at baseline.

In this paper, the authors have used a comparison of the imaging features of RIS according to whether individuals go on to develop a relapsing or primary progressive (PP) phenotype of MS, to provide insights into whether these disease subtypes should be regarded as separate entities. Increasingly, it is recognised that individuals entering a progressive phase of MS share many common characteristics, irrespective of whether they progress from the time of symptom-onset (PPMS) or following a relapsing-onset of symptoms (secondary progressive MS). This raises the possibility of a shared, common biology of focal inflammatory-demyelinating CNS lesions, and suggests that any distinction between these subtypes may be on arbitrary clinical grounds of whether any demyelinating lesion was symptomatic before the detection of progressive disability.

The authors describe the outcomes of a multi-centre RIS cohort of 453 patients. These individuals have been retrospectively identified but prospectively followed-up for clinical evidence of symptomatic CNS demyelination for durations ranging between 0.2 and 20 years. Baseline MRI features were analysed, in combination with clinical and demographic variables, to determine their value in predicting a relapsing versus primary progressive symptom-onset. Of the 453 patients, 128 developed symptoms in keeping with CNS inflammatory-demyelination. Of these, 113 (88 %) had a relapsing phenotype while 15 (12 %) had a primary progressive phenotype. Median time to conversion was 3.5 years in those who developed PPMS. The presence of at least one enhancing lesion and the presence of at least one spinal cord lesion were both more common in individuals who developed any form of symptomatic CNS demyelination. Subjects who evolved to PPMS were more likely to be male sex, older and to have at least one spinal cord lesion compared with those who had a relapsing-onset of symptoms. Perhaps surprisingly, there was no significant difference in the proportion of enhancing lesions between the relapsing- and progressive-onset groups. Neither was there any significant difference in time-to-conversion or CSF findings between those who developed relapsing versus progressive symptoms.

### Comment

The similarities presented here in the subclinical disease activity of individuals with a relapsing-onset and progressive-onset MS, supports the notion that the mechanisms of progression are likely to be similar in these two subgroups. The radiological predilection towards the spinal cord in PPMS is in keeping with the prevailing clinical phenotype in this subgroup. But it remains unexplained why radiological disease activity is less likely to manifest clinically as relapses in those with PPMS.

Studying the natural history of RIS prospectively at a population-level would pose a huge challenge of resources. Naturally, the randomness of case identification and absence of systematic follow-up in this study introduces potential for bias. But overall this study demonstrates the value of international collaborative endeavour in the study of rare diseases, or in this case, preclinical syndrome.

Kantarci et al. (2016) Ann Neurol 79:288–294

## Cortical atrophy patterns in multiple sclerosis are non-random and clinically relevant

The presence of grey matter pathology was documented in the earliest histopathological descriptions of MS. But grey matter pathology has received renewed interest in the last decade since demyelination of the grey matter was demonstrated to be widespread and to be a focus of neuro-axonal injury. Advanced MRI such as volumetry, diffusion-weighted imaging and magnetization-transfer imaging have all provided useful information on the extent and clinical relevance of grey matter pathology in MS. Grey matter atrophy has been shown to occur during the earliest stages of MS, but is most prominent in the later, progressive phase of the illness. Grey matter atrophy has been demonstrated to represent neuro-axonal loss and correlates more closely with physical and cognitive disability than any white matter imaging marker. But uncertainty remains over the cause of grey matter atrophy in MS and whether it is a diffuse process or adheres to a more regional pattern.

In this paper, the authors studied the pattern of cortical thickness in 208 patients with MS and 60 healthy controls. Using a novel technique called source based morphometry they sought to detect covariance in regional cortical thickness that was disseminated in space but related as part a functional network. Patients underwent detailed neuropsychological testing and physical examination to allow correlation of the clinical and imaging findings.

Results revealed ten discrete co-varying patterns of cortical thickness, of which six were more prominent in MS. Most of the patterns were non-random, symmetric and localized to known networks such as sensorimotor, limbic or default mode networks. As expected, global cortical thickness was lower in individuals with MS versus healthy controls. However, the presence of certain patterns of cortical atrophy showed more clinical relevance than global cortical atrophy. Higher EDSS was associated with cortical atrophy patterns including the bilateral sensorimotor and insular cortices. Combined with age and global cortical thickness, regional patterns explained 29 % of the variance in EDSS. Reduced cognitive ability was particularly associated discrete cortical atrophy patterns involving the limbic system and default mode network in a model explaining 43 % of the clinical variance. The addition of white matter imaging metrics into these models only marginally improved the explanation of clinical measures.

Those cortical atrophy patterns that differed most between MS and control participants were strongly associated with T2 lesion load but not with measures of normal-appearing white matter integrity. The relationships between white matter lesions and cortical atrophy differed according to clinical MS subgroups suggesting that some cortical regions remain more sensitive to white matter pathology throughout the entire course of MS.

### Comments

While imaging lacks the sensitivity or specificity of histopathology, it offers the enormous advantage of longitudinal study and insights into disease at all time-points. This paper illustrates how these strengths offer the potential to unravel some of the mysteries underlying CNS disease pathophysiology. The findings of this study led the authors to hypothesise that white matter demyelination initially drives cortical atrophy in anatomically connected regions. They suggest that neuronal loss within “cortical hubs” then leads to secondary (trans-synaptic) degeneration in other anatomically connected cortical regions across a network. One of the limitations of the paper, acknowledged by the authors, is that white matter demyelination was not defined on a network-level, only globally in the whole brain. It may be that future studies can further refine this hypothesis by relating pathological changes in all anatomical domains of a network, longitudinally within individuals.

Steenwijk et al. (2016) Brain 139:115–126

